# Dual latent tuberculosis screening with tuberculin skin tests and QuantiFERON-TB assays before TNF-α inhibitor initiation in children in Spain

**DOI:** 10.1007/s00431-022-04640-3

**Published:** 2022-11-05

**Authors:** Joan Calzada-Hernández, Jordi Anton, Javier Martín de Carpi, Berta López-Montesinos, Inmaculada Calvo, Ester Donat, Esmeralda Núñez, Javier Blasco Alonso, María José Mellado, Fernando Baquero-Artigao, Rosaura Leis, Ana María Vegas-Álvarez, Marta Medrano San Ildefonso, María del Carmen Pinedo-Gago, Francisco Javier Eizaguirre, Alfredo Tagarro, Marisol Camacho-Lovillo, Beatriz Pérez-Gorricho, César Gavilán-Martín, Sara Guillén, Belén Sevilla-Pérez, Luis Peña-Quintana, Pablo Mesa-Del-Castillo, Clàudia Fortuny, Marc Tebruegge, Antoni Noguera-Julian

**Affiliations:** 1 Pediatric Rheumatology Division, Hospital Sant Joan de Déu, Institut de Recerca Sant Joan de Déu, Barcelona, Spain; 2 Departament de Cirurgia i Especialitats Medicoquirúrgiques, Facultat de Medicina i Ciències de la Salut, Universitat de Barcelona, Barcelona, Spain; 3 Servei de Gastroenterologia, Hepatologia I Nutrició Pediàtrica, Hospital Sant Joan de Déu - Institut de Recerca Sant Joan de Déu, Barcelona, Spain; 4 Rheumatology Unit, Pediatrics Department, University Hospital La Fe, Valencia, Spain; 5 Pediatric Gastroenterology and Hepatology Unit, Pediatrics Department, University Hospital La Fe, Valencia, Spain; 6 UGC de Pediatría, Hospital Regional Universitario de Málaga, Málaga, Spain; 7 Servicio de Pediatria, Enfermedades Infecciosas Y Patología Tropical, Hospital La Paz, Madrid, Spain; 8 Red de Investigación Translacional en Infectología Pediátrica (RITIP), Madrid, Spain; 9 Centro de Investigación Biomédica en Red de Enfermedades Infecciosas (CIBERINFEC), Madrid, Spain; 10 Unit of Pediatric Gastroenterology, Hepatology and Nutrition, Pediatrics Department, University Clinical Hospital of Santiago (CHUS), Santiago de Compostela, Spain; 11 Gastroenterología Infantil, Hospital Universitario Río Hortega de Valladolid, Valladolid, Spain; 12 Reumatología Pediátrica, Hospital Universitario Miguel Servet, Saragossa, Spain; 13 Unidad de Reumatología Pediátrica, Hospital de Cruces, Barakaldo, Spain; 14 Unidad de Gastroenterología Infantil, Hospital Universitario Donostia, San Sebastián, Spain; 15 Paediatrics Department, Hospital Universitario Infanta Sofía; Paediatrics Research Group, Universidad Europea de Madrid, Madrid, Spain; 16 Fundación de Investigación Biomédica Hospital 12 de Octubre, Instituto de Investigación 12 de Octubre (imas12), Madrid, Spain; 17 Servicio de Inmunología, Reumatología e Infectología Pediátrica, Hospital Universitario Virgen del Rocío, Seville, Spain; 18 Pediatric Infectious Diseases Unit, Department of Pediatrics, Hospital Infantil Universitario Niño Jesús, Madrid, Spain; 19 Servicio de Pediatría, Hospital Universitario San Juan de Alicante, Alicante, Spain; 20 Department of Pediatrics, Hospital Universitario de Getafe, Madrid, Spain; 21 Unidad de Reumatología, Servicio de Pediatría del Hospital Universitario San Cecilio de Granada, Granada, Spain; 22 Pediatric Gastroenterology, Hepatology and Nutrition Unit, Mother and Child Insular University Hospital,, Las Palmas, Spain; 23 Centro de Investigación Biomédica en Red de Obesidad Y Nutrición (CIBEROBN), Madrid, Spain; 24 University Institute for Research in Biomedical and Health Sciences, University of Las Palmas de Gran Canaria, Las Palmas, Spain; 25 Department of Rheumatology, Hospital Clínico Universitario Virgen de La Arrixaca, Murcia, Spain; 26 Malalties Infeccioses I Resposta Inflamatòria Sistèmica en Pediatria, Unitat d’Infeccions, Servei de Pediatria, Institut de Recerca Sant Joan de Déu, Barcelona, Spain; 27 Centro de Investigación Biomédica en Red de Epidemiología Y Salud Pública (CIBERESP), Madrid, Spain; 28 Department of Infection, Immunity and Inflammation, UCL Great Ormond Street Institute of Child Health, University College London, London, UK; 29 Department of Paediatrics, University of Melbourne, Parkville, VIC Australia; 30 Department of Paediatrics, Klinik Ottakring, Wiener Gesundheitsverbund, Vienna, Austria

**Keywords:** Inflammatory bowel disease, Interferon-gamma release assay, Juvenile idiopathic arthritis, Pediatrics, Tuberculosis

## Abstract

Tumor-necrosis-factor-α inhibitors (anti-TNF-α) are associated with an increased risk of tuberculosis (TB) disease, primarily due to reactivation of latent TB infection (LTBI). We assessed the performance of parallel LTBI screening with tuberculin skin test (TST) and QuantiFERON-TB Gold In-Tube assays (QFT-GIT) before anti-TNF-α treatment in children with immune-mediated inflammatory disorders in a low TB-burden setting. We conducted a multicenter cohort study involving 17 pediatric tertiary centers in Spain. LTBI was defined as the presence of a positive TST and/or QFT-GIT result without clinical or radiological signs of TB disease. A total of 270 patients (median age:11.0 years) were included, mainly with rheumatological (55.9%) or inflammatory bowel disease (34.8%). Twelve patients (4.4%) were diagnosed with TB infection at screening (LTBI, *n* = 11; TB disease, *n* = 1). Concordance between TST and QFT-GIT results was moderate (TST+/QFT-GIT+, *n* = 4; TST−/QFT-GIT+, *n* = 3; TST+/QFT-GIT-, *n* = 5; kappa coefficient: 0.48, 95% CI: 0.36–0.60). Indeterminate QFT-GIT results occurred in 10 patients (3.7%) and were associated with young age and elevated C-reactive protein concentrations. Eleven of 12 patients with TB infection uneventfully completed standard LTBI or TB treatment. During a median follow-up period of 6.4 years, only 2 patients developed TB disease (incidence density: 130 (95% CI: 20–440) per 100,000 person-years), both probable de novo infections.

*Conclusion*: A substantial number of patients were diagnosed with LTBI during screening. The dual strategy identified more cases than either of the tests alone, and test agreement was only moderate. Our data show that in children in a low TB prevalence setting, a dual screening strategy with TST and IGRA before anti-TNF-α treatment is effective.

**Table:** 

**What is Known:** *• The optimal screening strategy for latent tuberculosis in children with immune-mediated inflammatory disorders remains uncertain.* *• Children receiving anti-TNF-α drugs are at increased risk of developing severe tuberculosis disease.*	
**What is New:** *• A dual screening strategy, using TST and an IGRA assay, identified more children with latent tuberculosis than either of the tests alone.* *• Identification and treatment of latent tuberculosis before initiation of anti-TNF-α therapy averted incident tuberculosis cases.*

**Supplementary Information:**

The online version contains supplementary material available at 10.1007/s00431-022-04640-3

## Introduction

Tumor necrosis factor-α (TNF-α) is a cytokine that plays a critical role in the formation and maintenance of granulomas, which are aggregates of macrophages and lymphocytes that contain pathogens that cannot be eliminated [1]. Upon primary infection with *Mycobacterium tuberculosis* (MTB), granulomas restrict the spread of MTB in the pulmonary parenchyma in most individuals, and the infection enters a stage of dormancy referred to as latent TB infection (LTBI) [1]. It has been estimated that approximately one-quarter of the world population has LTBI at present [2].

TNF-α is also implicated in the pathogenesis of several immune-mediated inflammatory disorders (IMID), including juvenile idiopathic arthritis (JIA) and inflammatory bowel disease (IBD), where high concentrations of TNF-α lead to chronic inflammation and tissue damage [3, 4]. TNF-α inhibitor therapy has changed the natural clinical course of many pediatric IMID patients, especially those with severe disease [5, 6]. Etanercept, infliximab, adalimumab, and golimumab are approved for the treatment of a range of IMID in children, and their use in other inflammatory conditions is currently being explored [7].

Patients receiving anti-TNF-α drugs are at substantially increased risk of developing mycobacterial infections, including TB disease [8]. Besides TNF-α inhibition, the IMID itself, the recent or concomitant use of other immunosuppressive drugs (especially corticosteroids), and co-existing comorbidities often contribute to increasing the risk of TB in this particular patient population. In adults on anti-TNF-α treatment TB disease usually results from LTBI reactivation, often manifesting as severe extrapulmonary or disseminated disease [7]. In contrast, in children treated with anti-TNF-α drugs TB disease typically stems from recent exposure and resulting uncontrolled primary infection [9].

The risk of LTBI reactivation has been reduced with the implementation of LTBI screening before the initiation of anti-TNF-α drugs and the provision of preventive treatment to those with positive test results [10]. Currently, there are only two types of diagnostic tests that can identify the presence of LTBI—the tuberculin skin test (TST) and interferon-gamma (IFN-γ) release assays (IGRAs). IGRAs are considered to be more specific than the TST, but have a number of disadvantages, including higher cost, the need for adequate laboratory facilities, and reduced accuracy in young children [11].

The optimal LTBI screening strategy in patients with IMID remains uncertain, and existing guidelines are based on low-quality evidence [12]. As with other immunocompromised individuals, many experts recommend a dual screening strategy, including both the TST and an IGRA test, to increase overall sensitivity [7]. However, there is considerable variation in the use of TST and IGRAs in widely used recommendations [12–15]. At present, in children and adolescents with IMID, LTBI screening recommendations prior to initiation of anti-TNF-α drugs are largely based on adult experience [16, 17].

This study aimed to assess the performance of parallel LTBI screening with TST and QuantiFERON-TB Gold In-Tube (QFT-GIT) assays before initiation of anti-TNF-α therapy in a large cohort of children and adolescents affected by IMID in a low TB burden setting.

## Materials and methods

### Setting and participating centers

We conducted a cohort observational multicenter study from November 2013 to September 2016 in Spain. In Spain, the incidence of TB has persistently decreased over the past 20 years to 9.3 cases/100,000 in 2019 (4.2/100,000 in individuals aged < 15 years) [18]. Bacillus Calmette-Guérin (BCG) vaccination is not part of the routine childhood immunization program in Spain, except for the Basque Country, where a single dose was given at birth until 2013. The Spanish Societies of Pediatric Rheumatology, Pediatric Gastroenterology and Pediatric Infectious Diseases, and the Spanish Pediatric TB Research Network [19, 20] provided support by encouraging their members to participate in this project. Ultimately, a total of 17 pediatric tertiary referral centers participated in the study.

### Eligibility criteria and case definitions

Children and adolescents (< 18 years-of-age at inclusion) affected by IMID in whom screening for LTBI was simultaneously performed the same day with TST and QFT-GIT before treatment with anti-TNF-α drugs was initiated were eligible for inclusion and recruited consecutively. Patients with a previous history of LTBI or TB disease, those previously treated with anti-TNFα drugs, and those with other underlying diseases that increase the risk of TB (malignancy, primary immunodeficiencies, and HIV infection) were excluded from participation. LTBI was defined as the presence of any positive result in the screening tests used (i.e., positive TST and/or QFT-GIT result) in the absence of clinical or radiological signs (if performed) of TB disease. The diagnosis of TB disease was based on epidemiological, clinical, radiological, and microbiological findings according to published consensus criteria [21], irrespective of TST or QFT-GIT results. In this manuscript, the term “TB infection” is used to encompass both LTBI and TB disease.

### Immunodiagnostic TB tests

TSTs were performed by intradermal injection of 0.1 mL (2 tuberculin units) of purified protein derivative (Tuberkulin PPD RT23, Statens Serum Institut, Copenhagen, Denmark), and results were read by trained personnel after 48–72 h. The cut-off for a positive TST result was defined according to national guidelines as an induration ≥ 5 mm, independent of BCG vaccination status [22]. All QFT-GIT assays were performed in fully accredited routine diagnostic laboratories at each participating center and interpreted according to manufacturer’s instructions [23]. In brief, the assay result was classified as positive if the concentration of IFN-γ in the antigen-stimulated sample was ≥ 0.35 IU/mL after subtraction of the IFN-γ concentration in the negative control sample, irrespective of the IFN-γ concentration in the positive control sample. The result was classified as negative if the background-corrected IFN-γ concentration in the antigen-stimulated sample was < 0.35 IU/mL with adequate response in the mitogen-stimulated sample (positive control ≥ 0.50 IU/mL). If the background-corrected IFN-γ concentration in the antigen-stimulated sample was < 0.35 IU/mL with a positive control response < 0.50 IU/mL or the IFN-γ concentration in the negative control was ≥ 8.0 IU/mL, the result was classified as indeterminate.

### Data collection

A standardized data collection tool was designed and distributed to all co-investigators. The returned data were collated into an Excel database (Microsoft, Redmond, WA) hosted on a secure server. The following variables were collected: demographics (gender, age at IMID diagnosis, age at LTBI screening, country of birth, ethnicity), type of IMID, immunosuppressive treatment in the previous 3 months (corticosteroids, disease-modifying antirheumatic drugs (DMARD) or combined corticosteroids and DMARD), BCG vaccination status based on presence of a visible scar in the deltoid region and/or a positive history, history of TB contact, TST (in mm induration) and QFT-GIT results (positive, negative, or indeterminate), chest X-ray (CXR) findings, erythrocyte sedimentation rate (ESR; normal value < 15 mm), and C-reactive protein (CRP; normal value < 15 mg/L) levels. In patients diagnosed with LTBI or TB disease and those with indeterminate QFT-GIT results, additional information was requested from the co-investigator about further diagnostic workup, final diagnosis regarding TB, TB treatment regimen, adverse events related to TB drugs, outcome of TB infection, and subsequent anti-TNF-α treatment initiation. In the remaining of patients, co-investigators were asked to report the duration of follow-up after LTBI screening and whether or not the patient was subsequently diagnosed with TB.

### Statistical methods

Quantitative variables are reported as medians and interquartile ranges (IQR) and categorical variables as proportions with 95% confidence intervals (95% CI). Bivariate analysis was performed using chi-square and Fisher’s exact tests for categorical variables, and Student’s *t* and ANOVA tests for quantitative variables. Non-normally distributed variables were compared with Mann–Whitney *U* and Kruskal–Wallis tests. Binary logistic regression modeling, in which all variables with a *p*-value < 0.1 in bivariate analysis were included, was used for multivariate analysis to test the effect of covariates as risk factors for presence versus absence of TB infection, and for determinate (i.e., positive or negative) versus indeterminate QFT-GIT results. The results are presented as adjusted odds ratios (aOR) with 95%CIs.

Because there is no universally agreed gold standard for LTBI diagnosis, and both TST and QFT-GIT were therefore included in the study case definition for LTBI, we were unable to determine the sensitivity and the negative predictive value of those tests, but instead evaluated the concordance between the tests. Total percentage agreement and Cohen kappa coefficient (*κ*) were used to quantify concordance between TST and QFT-GIT results; indeterminate QFT-GIT results were excluded from this particular analysis. Strength of agreement was defined as poor (*κ* ≤ 0.2), fair (0.2 < *κ* ≤ 0.4), moderate (0.4 < *κ* ≤ 0.6), good (0.6 < *κ* ≤ 0.8), and excellent (*κ* > 0.8). All statistical analyses were performed using SPSS V24 (IBM; Armond, NY), with statistical significance defined as a *p*-value < 0.05.

### Ethics approval

The study was carried out in accordance with the Declaration of Helsinki. Ethics approval was obtained from the Hospital Sant Joan de Déu Ethics Committee (reference PIC 133-13) and from the ethics committees of every participating center thereafter. Informed consent for participation was obtained from the parents/guardians of each participant and assent in patients aged > 12 years.

## Results

**Fig. 1:**
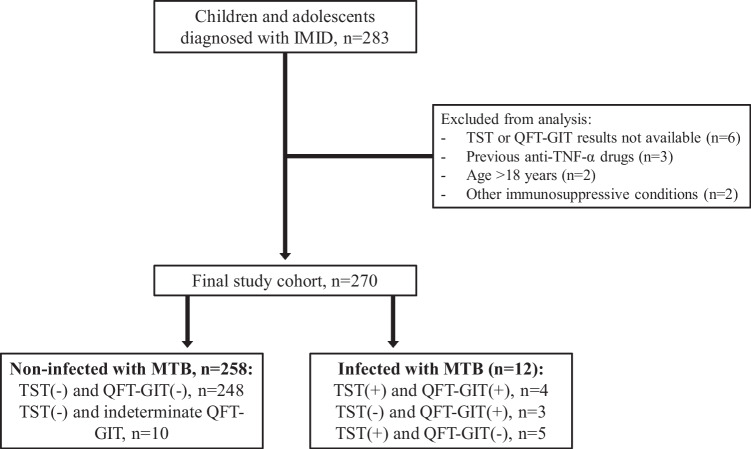
Flowchart showing the patients screened and included, and the final diagnosis regarding MTB infection according to the immunologic tests that were used in this study

Overall, 283 patients (163 girls, 57.6%) were submitted by the participating centers, 13 of whom did not fulfill the inclusion criteria (Fig. 1). Therefore, a total of 270 patients were included in the final study population (153 girls, 56.7%). The predominant underlying IMID comprised JIA (52.2%), IBD (Crohn’s disease 23.7%; ulcerative colitis 11.1%), and idiopathic uveitis (7.4%) (Supplementary Table 1). Spain was the most common country of family origin (88.1%) and also the commonest country of birth (93.7%). Twenty-four patients (8.9%) had previously been vaccinated with BCG; 18 of those were born in Spain.

Median (IQR) age at IMID diagnosis and at LTBI screening was 8.6 (3.8–12.0) and 11.0 (6.5–13.2) years, respectively. At the initial assessment, none of the patients reported previous contact with a TB patient. In the preceding 3 months, 104 (38.5%), 22 (8.1%), and 79 (29.3%) of the participants had been treated with conventional DMARDs (other than anti-TNF-α drugs), corticosteroids, or both, respectively; only 65 (24.1%) had not received any immunosuppressant drugs during that period.

CXRs were performed in 190 (70.4%) patients, and radiological findings consistent with TB were observed in only one participant (patient 10, Table 1). Median (IQR) ESR and CRP levels were 14 (5–28) mm and 2.9 (0.7–15.9) mg/L, respectively; 51.7% (135/261) and 25.4% (68/268) of the patients had elevated ESR or CRP levels, respectively.

**Table 1:** Summary of epidemiological and clinical details in patients diagnosed with TB infection. Details on demographics and clinical characteristics, immunodiagnostic test results at initial assessment, anti-TB treatment regimens, and follow-up outcomes in patients diagnosed with TB infection

	Gender, age at assessment (years)	BCG vaccination status	IMID	Immunosuppressive treatment in preceding 3 months	TST result (induration in mm)/QFT-GIT result (IU/mL)^a^	Chest X-ray findings	Anti-TB treatment, adverse events	Anti-TNF-α and other biologic drugs (age at initiation in years)	Incident TB disease/age at most recent follow-up (years)
1	M, 6.9	Non-vaccinated	JIA	None	Pos (12)/Neg (0.04)	Unremarkable	H for 9 months, none	ETN (7.2)	No/13.0
2	F, 8.5	Non-vaccinated	JIA	None	Pos (15)/Pos (4.78)	Unremarkable	HR for 3 months, none	ETN (9.2), ADA (13.6), tocilizumab (15.0)	No/16.0
3 [24]	F, 9.3	Non-vaccinated	JIA	CS, MTX	Pos (10)/Pos (NA)	Unremarkable	H for 9 months, none	ETN (10.9), ADA (12.0)	Yes (12.2)/14.7
4	M, 10.3	Non-vaccinated	JIA	MTX	Neg (0)/Pos (NA)	Unremarkable	H for 9 months, none	ETN (10.5), ADA (13.8)	No/16.3
5	F, 10.6	Non-vaccinated	JIA	MTX	Pos (9)/Neg (0.00)	Unremarkable	HR for 3 months, none	None	No/15.2
6	F, 14.3	Vaccinated	JIA	CS, MTX	Pos (10)/Neg (0.00)	Unremarkable	HR for 3 months, none	ETN (14.4), tocilizumab (15.2), rituximab (16.3)	No/18.2
7	F, 14.8	Vaccinated	JIA	MTX, anti-IL-6	Pos (15)/Pos (NA)	Unremarkable	H for 9 months, mild self-limiting nausea	Canakinumab (15.2)	No/19.6
8	M, 15.9	Non-vaccinated	JIA	CS, MTX	Pos (6)/Neg (NA)	Not performed	No anti-TB treatment	ADA (16.1)	No/20.8
9	M, 17.3	Vaccinated	JIA	None	Pos (20)/Pos (11.37)	Unremarkable	H for 6 months, none	ETN (NA)	No/18.1
10	M, 10.2	Non-vaccinated	Systemic sclerosis	None	Neg (0)/Pos (9.96)	Fibroparenchymal opacities and a calcified right hilar lymph node	HRZE for 2 months and HR for 4 months, none	None	No/14.9
11	F, 13.7	Non-vaccinated	Crohn’s disease	CS	Neg (0)/Pos (NA)	Unremarkable	H for 9 months, none	IFX (13.9)	No/18.6
12	M, 10.8	Vaccinated	Idiopathic uveitis	None	Pos (10)/Neg (NA)	Unremarkable	R for 4 months, none	None	No/15.8

*ADA* adalimumab, *CS* corticosteroids, *E* ethambutol, *ETN* etanercept, *F* female, *H* isoniazid, *IL-6* interleukin 6, *IFX* infliximab, *M* male, *MTX* methrotexate, *NA* not available, *Neg* negative, *Pos* positive, *R* rifampicin, *Z* pyrazinamide

^a^IFN-γ concentration in QFT-GIT antigen tube minus IFN-γ concentration in nil control tube

Overall, 12 patients (4.4%; 10 patients [3.7%] if a 10 mm TST cut-off had been used) were found to have TB infection at the initial assessment, comprising 11 patients with LTBI and one with TB disease. The latter was a 10-year-old boy who had systemic sclerosis but no TB symptoms at assessment; he had a negative TST, but a positive QFT-GIT result; CXRs showed fibro parenchymal opacities and a calcified right hilar lymph node (patient 10, Table 1). Among patients with TB infection, TST and IGRA result constellations varied widely (Table 1): TST+/QFT-GIT+, *n* = 4; TST−/QFT+, *n* = 3; and TST+/QFT-GIT−, *n* = 5. In bivariate analysis, only prior BCG vaccination (16.7% vs. 2.9% in unvaccinated patients; *p* = 0.012), non-Caucasian ethnicity (17.9% vs. 3.0% in Caucasian patients; *p* = 0.009), and birth abroad (33.3% vs. 2.7% in patients born in Spain; *p* < 0.0001) were associated with an increased risk of TB infection. In the logistic regression model, only birth abroad remained associated with an elevated risk of TB infection (aOR 20.7 (95% CI 1.3–333.2); *p* = 0.032).

In the whole cohort, after excluding indeterminate QFT-GIT results, concordance between TST and QFT-GIT results was only moderate (96.9%; *k* = 0.484, 95% CI 0.36–0.60). Indeterminate QFT-GIT results were observed in 10 patients (3.7%); all were due to insufficient responses in the positive control (mitogen) tube (Supplementary Table 2). All 10 patients had a negative TST result and an unremarkable CXR. In 4 of these patients, further TB immunodiagnostic tests were performed and yielded negative results. In bivariate analysis, a trend toward increased rates of indeterminate QFT-GIT results was observed with younger age at initial assessment (median: 7.9 years vs. 11.1 years in patients with a determinate QFT-GIT result; *p* = 0.095). Also, patients with indeterminate results had higher median CRP (30.8 vs. 2.9 mg/L; *p* = 0.027) and ESR (32 vs. 14 mm; *p* = 0.094) levels than those with determinate assay results. In the logistic regression model, statistical significance was maintained for younger age at initial assessment (aOR 0.838 (95% CI 0.708–0.993) per year of age increase; *p* = 0.042) and higher CRP levels (aOR 1.019 (95% CI 1.001–1.037) per 1 mg/L increase; *p* = 0.043).

Eleven out of 12 patients with TB infection received standard LTBI (*n* = 10) or TB (*n* = 1) treatment regimens [22], without major adverse events (Table 1); in the remaining patient (patient 12), a decision was made not to initiate preventive therapy, but to use close monitoring instead. Eight of the 11 patients who received anti-TB therapy were subsequently commenced on anti-TNFα treatment 0.1–1.6 years after the initial assessment. Only one patient, a 9-year-old girl with JIA (patient 3, Table 1) who was treated with a 9-month isoniazid regimen and then sequentially received etanercept and adalimumab, developed disseminated TB disease 3 years after initial assessment. The history revealed that she had recently had contact with a relative with pulmonary TB. Typing of her MTB isolates confirmed complete homology with the strain isolated from her relative, indicating that she had de novo infection rather than reactivation of LTBI [24]. Anti-TNF-α drugs were also started in 9 out of 10 patients with indeterminate QFT-GIT results (ranging from 0 to 1.6 years after assessment), none of whom developed TB disease during follow-up (Supplementary Table 2). Of the remaining cohort (all TST−/QFT−), only a 15-year-old boy born in Morocco who had Crohn’s disease was diagnosed with unconfirmed pulmonary TB while receiving adalimumab, 5 years after baseline assessment. He reported traveling twice to Morocco after the initial screening; no other risk factors for TB infection were identified. At TB diagnosis, repeat TST and a QuantiFERON-TB Gold Plus assay were negative. He showed good clinical and radiological response to 6-month standard anti-TB treatment. Overall, after a median follow-up period of 6.4 years, the incidence density of TB disease in this cohort was 130 (95% CI: 20–440) per 100,000 person-year.

## Discussion

Our study shows that a dual LTBI screening strategy using both TST and an IGRA assay in children and adolescents prior to initiation of anti-TNF-α treatment is effective. Even though Spain is a low TB prevalence country, close to 1 in 20 patients had at least one positive immunodiagnostic test result at baseline screening, suggesting they had LTBI. Crucially, detection of LTBI in those individuals facilitated targeted preventive therapy to avert future progression to TB disease. During a median follow-up of more than 6 years, only two (0.7%) of the 270 study participants subsequently developed TB disease, equating to an observed incidence density of 130 per 100,000 person-year. In the first of those patients (the child with JIA), the history and laboratory findings make de novo infection years after LTBI screening, rather than reactivation of LTBI, highly likely. The second patient (with Crohn’s disease) presented more than 5 years after LTBI screening. This substantial delay indicates that this was also likely to be a de novo infection, as published data show that LTBI reactivation almost invariably occurs within the first 2 years of starting anti-TNF-α treatment [9].

Importantly, TB disease in children receiving anti-TNF-α therapy almost universally manifests as severe and often disseminated disease. In the largest pediatric case series to date, which comprises a total of 19 patients treated with anti-TNF-α drugs who developed TB disease, all patients presented with pre-defined severe disease, and one was diagnosed only at a post-mortem examination [9]. Of the 18 patients who were alive at presentation, 14 had miliary TB, and 4 had central nervous system involvement, both of which are associated with high levels of morbidity and mortality [25, 26]. This predilection for severe TB manifestations in children on anti-TNF-α treatment is further documented by several case reports and small case series [27, 28]. Notably, the aforementioned study also showed those children typically require prolonged TB treatment (median duration: 50 weeks), and that the risk of long-term sequelae in this patient population is substantial (observed in 28% of the survivors).

The growing number of indications of anti-TNF-α agents in the pediatric age group, the rising incidence of IBD in newly-industrialized countries [29], the availability of affordable and therapeutically equivalent biosimilars, and the recent inclusion of anti-TNF-α drugs into the WHO Essential Medicines for Children List for the treatment of JIA and Crohn’s disease (available at: https://list.essentialmeds.org/) make a sustained global increase in the use of those drugs in children and adolescents likely, including in regions with high TB prevalence. Ultimately, this may result in a greater proportion of pediatric TB patients presenting with severe TB disease than previously [30].

We used a simultaneous dual LTBI screening strategy in our study, as recommended by most recent guidelines for this particular patient population [7, 15, 17]. Concordant negative results were required to exclude TB infection in those very high-risk children. IGRA assays have been shown to have a high negative predictive value in previously healthy children [31, 32], but whether this is also the case in children with IMID, including those who have not commenced immunosuppressive therapy yet, remains uncertain. Our data highlight that discordance between TST and IGRA results is common in children with IMID, with only few patients showing concordantly positive test results. This indicates that the LTBI detection yield would be substantially lower if only one of those tests was used for screening. Similar observations regarding high levels of result discordance were reported by adults [33, 34] and smaller pediatric studies (Table 2). It could be argued that in some BCG-vaccinated patients, a TST+/IGRA− result constellation may simply reflect a false-positive TST result caused by the vaccine. However, there are increasing data to suggest that a large proportion of those children do in fact have LTBI (i.e., true-positive TST and false-negative IGRA results) [39]. Given that in routine practice, there is currently no modality to distinguish between those two groups, and considering that preventive therapy is generally well-tolerated by children while the risk of severe, potentially fatal TB disease if progression occurs is substantial, we and other experts recommend to provide preventive treatment to children with a TST+/IGRA− result constellation [40].

**Table 2:** Overview of studies evaluating TST and IGRAs in children with IMID. Only published pediatric studies assessing the performance of TST and IGRAs in LTBI screening in children and adolescents affected by IMID before the implementation of anti-TNF-α that included more than 10 participants and provide a direct head-to-head comparison between TST and a commercial IGRA performed simultaneously were included

**Reference**	**Country**	**Type of IMID (no. of participants)**	**Proportion of BCG-vaccinated participants**	**Immunodiagnostic TB tests used** **(TST cut-off)**	**Concordance (%)**	**Cohen kappa coefficient**	**Proportion of indeterminate IGRA results**
[35]	Turkey	JIA (*n* = 39)	74.3%	TST (10 mm) vs. QFT-GIT	66.7%	−0.095	0%
[36]	Czech Republic	IBD (*n* = 46)	100%	TST (ND) vs. QFT-GIT	97.3%	*	17.4%
[37]	Italy	JIA (*n* = 120)	ND	TST (10 mm) vs. QFT-Gold	96.5%	0.483	3.3%
[38]	Turkey	Rheumatological IMID (*n* = 57)	100%	TST (5 mm) vs. T-SPOT. *TB*	73.7%	0.176	0%
Current study	Spain	Various IMID (*n* = 270)	8.9%	TST (5 mm) vs. QFT-GIT	96.9%	0.484	3.7%

*ND* no data, *QFT-Gold* QuantiFERON-TB Gold

^*^Cohen kappa coefficient is noncalculable, as the study did not include any patients with a TST+/QFT-GIT+ result constellation

The rate of indeterminate QFT-GIT results in our study was 3.7%, similar to the 4% rate reported by a recent meta-analysis including more than 100,000 children [41]. All indeterminate results in our cohort were due to insufficient positive control responses. In multivariate analysis, young age and increased CRP levels at assessment were associated with a higher risk of indeterminate results. This aligns with previous data showing that IGRAs perform less well at the extremes of age [42]. Furthermore, we have recently reported that both elevated CRP levels and lymphopenia are associated with indeterminate QFT Gold Plus results in a large cohort of predominantly healthy children and adolescents [43]. Underlying IMID activity has also been associated with indeterminate results, both when assessed by activity scores or by laboratory parameters [36]. Three-quarters of the patients in our cohort were already receiving conventional DMARDs and/or corticosteroids at the initial assessment; however, we did not observe a statistically significant association between previous treatment and IGRA performance in our study. Nevertheless, exposure to corticosteroids, anti-TNF-α drugs, and other immunomodulators, including cyclosporin, has previously been shown to have the potential to impair the performance of IGRAs substantially and to increase the proportion of indeterminate and also false-negative assay results [44, 45]. Therefore, initial LTBI screening should be performed at the point when an IMID diagnosis is made, before initiation of immunosuppressive treatment. In agreement with most guidelines [12, 15, 17], we also believe that initial LTBI screening should include a chest X-ray to identify radiographic evidence of prior TB, as it happened in one patient in our study.

Our observations also highlight the need to integrate specific questions about potential recent TB exposure and new risk factors for TB (e.g., travel to high TB prevalence region) into regular clinical reviews while patients are receiving anti-TNF-α agents, considering that one of the study participants who developed TB disease had known TB exposure. Furthermore, parents should be made aware of the elevated risk of TB disease and instructed to seek medical attention if a persistent cough or constitutional symptoms occur. Physicians should have a low threshold to initiate a detailed TB workup in patients with symptoms that could be consistent with TB. Currently, the role of regular LTBI screening in an asymptomatic child on anti-TNF-α therapy without new risk factors remains uncertain [46].

Our study has a number of limitations, including the absence of a universally agreed gold standard for LTBI, shared with all other immunodiagnostic TB studies. Furthermore, LTBI screening tests were not routinely repeated during follow-up, and we could not assess any information about potential result conversions and reversions. Also, we were not able to collect quantitative QFT-GIT results, which may have provided additional insights. Finally, our results cannot necessarily be extrapolated to the latest generation QFT assay, QuantiFERON-TB Gold Plus; however, an increasing number of studies suggest that its performance does not differ substantially from previous-generation QFT assays [43].

In conclusion, by use of a dual LTBI screening strategy with TST and IGRA performed in parallel, close to 1 in 20 children who were planned to be commenced on anti-TNF-α agents were found to have evidence of LTBI, despite the low TB prevalence setting. Result concordance between both tests was only moderate, highlighting that the detection yield would be substantially lower if only a single immunodiagnostic test was to be used. Identification of children with LTBI allowed targeted preventive therapy before initiation of anti-TNF-α therapy to avert future progression to TB disease. Only two children developed TB disease during follow-up. In both de novo infection was far more likely to be the cause than LTBI reactivation, indicating that the screening and treatment strategies employed in this study were effective.

## Supplementary Information

Below is the link to the electronic supplementary material.

Supplementary file1 (PDF 42 KB)

## Data Availability

The data underlying this article will be shared on reasonable request to the corresponding author.

## Abbreviations

anti-TNF-α: Tumor-necrosis-factor-α inhibitors
aOR: Adjusted odds ratio
BCG: Bacillus Calmette-Guérin
CI: Confidence interval
CRP: C-reactive protein
CXR: Chest X-ray
DMARD: Disease-modifying antirheumatic drugs
ESR: Erythrocyte sedimentation rate
IBD: Inflammatory bowel disease
IGRAs: Interferon-gamma release assays
IMID: Immune-mediated inflammatory disorders
IFN-γ: Interferon-gamma
IQR: Interquartile range
JIA: Juvenile idiopathic arthritis
κ: Cohen kappa coefficient
LTBI: Latent tuberculosis infection
MTB: *Mycobacterium tuberculosis*
QFT-GIT: QuantiFERON-TB Gold In-Tube
TB: Tuberculosis
TST: Tuberculin skin test
