# Supplementary material for: Dual latent tuberculosis screening with tuberculin skin tests and QuantiFERON-TB assays before TNF-α inhibitor initiation in children in Spain
Source: Eur J Pediatr. 2022 Nov 5;182(1):307–17. doi: 10.1007/s00431-022-04640-3 (PMC9829583; doi:10.1007/s00431-022-04640-3)
Supplement: Supplementary file 1 — Supplementary file1 (PDF 42 KB) [file 431_2022_4640_MOESM1_ESM.pdf]

European Journal of Pediatrics

Dual latent tuberculosis screening with tuberculin skin tests and QuantiFERON-TB assays before TNF-α inhibitor initiation in children in Spain

Joan CALZADA-HERNÁNDEZ, MD (joan.calzada@sjd.es); Unitat de Reumatologia Pediàtrica, Servei de Pediatria, Hospital Sant Joan de Déu - Institut de Recerca Sant Joan de Déu; Barcelona, Spain

Jordi ANTON, MD, PhD (jordi.anton@sjd.es); Unitat de Reumatologia Pediàtrica, Servei de Pediatria, Hospital Sant Joan de Déu - Institut de Recerca Sant Joan de Déu; Barcelona, Spain. Departament de Pediatria, Universitat de Barcelona; Barcelona, Spain.

Javier MARTÍN DE CARPI, MD, PhD (javier.martinc@sjd.es); Departament de Pediatria, Universitat de Barcelona; Barcelona, Spain. Servei de Gastroenterologia, Hepatologia i Nutrició Pediàtrica, Hospital Sant Joan de Déu - Institut de Recerca Sant Joan de Déu; Barcelona, Spain.

Berta LÓPEZ-MONTESINOS, MD (berlomon@yahoo.com); Rheumatology Unit, Pediatrics Department, University Hospital La Fe; Valencia, Spain.

Inmaculada CALVO, MD, PhD (calvo_inm@gva.es); Rheumatology Unit, Pediatrics Department, University Hospital La Fe; Valencia, Spain.

Ester DONAT, MD, PhD (donat_est@gva.es); Pediatric Gastroenterology and Hepatology Unit, Pediatrics Department, University Hospital La Fe; Valencia, Spain.

Esmeralda NÚÑEZ, MD, PhD (esmenunez@gmail.com); UGC de Pediatría, Hospital Regional Universitario de Málaga; Málaga, Spain.

Javier BLASCO ALONSO, MD, PhD (javierblascoalonso@yahoo.es); UGC de Pediatría, Hospital Regional Universitario de Málaga; Málaga, Spain.

María José MELLADO, MD, PhD (mariajose.mellado@salud.madrid.org); Servicio de Pediatria, Enfermedades Infecciosas y Patología Tropical, Hospital La Paz; Madrid, Spain. Red de Investigación Translacional en Infectología Pediátrica (RITIP); Madrid, Spain. Centro de Investigación Biomédica en Red de Enfermedades Infecciosas (CIBERINFEC); Madrid, Spain

Fernando BAQUERO-ARTIGAO, MD, PhD (fbaqueroartigao@gmail.com); Servicio de Pediatria, Enfermedades Infecciosas y Patología Tropical, Hospital La Paz; Madrid, Spain. Red de Investigación Translacional en Infectología Pediátrica (RITIP); Madrid, Spain. Centro de Investigación Biomédica en Red de Enfermedades Infecciosas (CIBERINFEC); Madrid, Spain

Rosaura LEIS, MD, PhD (mariarosaura.leis@usc.es); Unit of Pediatric Gastroenterology, Hepatology and Nutrition, Pediatric Department, University Clinical Hospital of Santiago (CHUS); Santiago de Compostela, Spain.

Ana María VEGAS-ÁLVAREZ, MD (ANAVEGAS@telefonica.net); Gastroenterología Infantil, Hospital Universitario Río Hortega de Valladolid; Valladolid, Spain.

Marta MEDRANO SAN ILDEFONSO, MD (mmedrano@unizar.es); Reumatología Pediátrica. Hospital Universitario Miguel Servet; Zaragoza, Spain.

María del Carmen PINEDO-GAGO, MD (mariadelcarmen.pinedogago@osakidetza.net); Unidad de Reumatología Pediátrica, Hospital de Cruces; Barakaldo, Spain.

Francisco Javier EIZAGUIRRE-AROCENA, MD, PhD (FRANCISCOJAVIER.EIZAGUIRREAROCENA@osakidetza.net); Unidad de Gastroenterología Infantil, Hospital Universitario Donostia; San Sebastián, Spain.

Alfredo TAGARRO, MD, PhD (alfredotagarro@hotmail.com); Red de Investigación Translacional en Infectología Pediátrica (RITIP); Madrid, Spain. Paediatrics Department, Hospital Universitario Infanta Sofía; Paediatrics Research Group, Universidad Europea de Madrid; Madrid, Spain. Fundación de Investigación Biomédica Hospital 12 de Octubre, Instituto de Investigación 12 de Octubre (imas12); Madrid, Spain.

Marisol CAMACHO-LOVILLO, MD (marisolcl73@gmail.com); Servicio de Inmunología, Reumatología e Infectología pediátrica, Hospital Universitario Virgen del Rocío; Sevilla, Spain.

Beatriz PÉREZ-GORRICHO, MD, PhD (bpgorricho@salud.madrid.org); Pediatric Infectious Diseases Unit, Department of Pediatrics, Hospital Infantil Universitario Niño Jesús; Madrid, Spain.

César GAVILÁN-MARTÍN, MD, PhD (cesargavilan@hotmail.com); Servicio de Pediatría, Hospital Universitario San Juan de Alicante; Alicante, Spain.

Sara GUILLÉN, MD, PhD (sguillenmartin@hotmail.com); Department of Pediatrics, Hospital Universitario de Getafe, Madrid, Spain. Centro de Investigación Biomédica en Red de Enfermedades Infecciosas (CIBERINFEC); Madrid, Spain.

Belén SEVILLA-PÉREZ, MD (belensev@hotmail.com); Unidad de Reumatología, Servicio de Pediatría del Hospital Universitario San Cecilio de Granada; Granada, Spain.

Luis PEÑA-QUINTANA, MD, PhD (lpena@dcc.ulpgc.es); Pediatric Gastroenterology, Hepatology and Nutrition Unit, Mother and Child Insular University Hospital, Las Palmas, Spain. Centro de Investigación

Biomédica en Red de Obesidad y Nutrición (CIBEROBN); Madrid, Spain. University Institute for Research in Biomedical and Health Sciences, University of Las Palmas de Gran Canaria; Las Palmas, Spain.

Pablo MESA-DEL-CASTILLO, MD (mesacasti@gmail.com); Department of Rheumatology, Hospital Clínico Universitario Virgen de la Arrixaca; Murcia, Spain.

Clàudia FORTUNY, MD, PhD (cfortuny@hsjdbcn.es); Malalties Infeccioses i Resposta Inflamatòria Sistèmica en Pediatria, Unitat d'Infeccions, Servei de Pediatria, Institut de Recerca Sant Joan de Déu; Barcelona, Spain. Centro de Investigación Biomédica en Red de Epidemiología y Salud Pública (CIBERESP); Madrid, Spain. Red de Investigación Translacional en Infectología Pediátrica (RITIP); Madrid, Spain. Departament de Pediatria, Universitat de Barcelona; Barcelona, Spain.

*Marc TEBRUEGGE, DTM&H, DLSHTM, MRCPCH, MSc, FHEA, MD, PhD (m.tebruegge@ucl.ac.uk); Department of Infection, Immunity and Inflammation, UCL Great Ormond Street Institute of Child Health, University College London, London, United Kingdom. Department of Paediatrics, University of Melbourne, Parkville, Victoria, Australia.

*Antoni NOGUERA-JULIAN, MD, PhD (ton@hsjdbcn.es); Malalties Infeccioses i Resposta Inflamatòria Sistèmica en Pediatria, Unitat d'Infeccions, Servei de Pediatria, Institut de Recerca Sant Joan de Déu; Barcelona, Spain. Centro de Investigación Biomédica en Red de Epidemiología y Salud Pública (CIBERESP); Madrid, Spain. Red de Investigación Translacional en Infectología Pediátrica (RITIP); Madrid, Spain. Departament de Pediatria, Universitat de Barcelona; Barcelona, Spain.

on behalf of the TST vs IGRA Study Investigators (see Study Group).

(*) Both authors contributed equally.

Corresponding author:

Antoni NOGUERA-JULIAN, MD, PhD

Hospital Sant Joan de Déu

Passeig Sant Joan de Déu 2, 08950 Esplugues, Spain

Phone number: +34 93 280 40 00 (ext. 80063); fax number: +34 93 203 39 59

E-mail address: ton@hsjdbcn.es; ORCID iD: 0000-0001-7485-0583

Supplementary Table 1. **Baseline characteristics of the patients included in the study.** **U**nderlying IMID and epidemiologic details of the 270 patients included in the study.

**Characteristics**	
**Female sex, n (%)**	153 (56.7)
**Age at IMID diagnosis, years [median (IQR)]**	8.6 (3.8-12.0)
**Age at TB Infection screening, years [median (IQR)]**	11.0 (6.5-13.2)
Patients <5 years of age, n (%)	49 (18.1)
**Immune-mediated inflammatory disease, n (%)**	
Juvenile idiopathic arthritis	141 (52.2)
Inflammatory bowel disease	
Crohn´s disease	64 (23.7)
Ulcerative colitis	30 (11.1)
Idiopathic uveitis	20 (7.4)
Other conditions:	15 (5.6)
Systemic lupus erythematosus	2
Recurrent idiopathic pericarditis	2
Panarteritis nodosa	2
Behçet's disease	2
Systemic sclerosis	1
Psoriasis	1
Linear scleroderma	1
Juvenile dermatomyositis	1
Autoimmune hepatitis	1
Autoimmune enteropathy	1
Synovitis, acne, pustulosis, hyperostosis and osteitis syndrome	1
**Country of birth, n (%)**	
Spain	253 (93.7)
Other countries:	17 (6.3)
Morocco	6
Andorra	2
Colombia	2
Romania	2
Algeria	1
China	1
Ecuador	1
Moldova	1
Ukraine	1
**Family origin, n (%)**	
Western Europe (including Spain)	240 (88.9)
North Africa	15 (5.6)
Eastern Europe	7 (2.5)
Latin America	6 (2.2)
Asia	1 (0.4)
Subsaharan Africa	1 (0.4)

Supplementary Table 2. **Summary of epidemiological and clinical details in patients with indeterminate QFT-GIT results.** Details on demographics, clinical characteristics, preceding immunosuppressive treatment, laboratory results, and follow-up outcome in the 10 patients who had indeterminate QFT-GIT results at the initial assessment.

	Gender, age at assessment (years)	BCG vaccination status	IMID	Immunosuppressive treatment in preceding 3 months	ESR (mm) / CRP (mg/L)	Further TB immunodiagnostic tests performed after the initial assessment	Anti-TNF-α and other biologic drugs (age at initiation in years)	Incident TB disease / age at most recent follow-up (years)
1	F, 1.2	Non-vaccinated	JIA	CS and CsA	113 / 65.8	Repeat QFT-GIT negative (after 20 months)	Anakinra (1.4) and canakinumab (3.2)	No / 5.7
2	F, 2.5	Non-vaccinated	JIA	MTX	32 / 23.7	TST 0mm, indeterminate QFT-GIT, T-SPOT.*TB* negative (after 4 weeks)	ADA (2.6)	No / 8.0
3	M, 4.1	Non-vaccinated	JIA	CS and MTX	16 / 15.0	Repeat QFT-GIT negative (after 2 months)	ETN (4.1), ADA (4.5), abatacept (5.6), IFX (6.3) and tofacitinib (7.5)	No / 9.1
4	M, 4.7	Vaccinated	JIA	MTX	68 / 59.0	None	ETN (5.0)	No / 8.8
5	M, 7.4	Non-vaccinated	JIA	None	49 / 91.0	Repeat QFT-GIT negative (after 12 months)	ETN (8.3)	No / 12.6
6	M, 11.2	Non-vaccinated	Crohn´s disease	CS and AZA	NA / 0.6	None	ADA (11.5), vedolizumab (15.3) and risankizumab (16.2)	No / 16.6
7	M, 12.1	Non-vaccinated	Crohn´s disease	None	30 / 82.4	None	ADA (12.1)	No / 16.0
8	F, 8.3	Non-vaccinated	Ulcerative colitis	CS	37 / 37.8	None	IFX (8.3)	No / 13.3
9	M, 13.2	Non-vaccinated	Ulcerative colitis	CS	2 / 2.1	None	IFX (15.2)	No / 17.6
10	M, 12.1	Non-vaccinated	Idiopathic uveitis	MTX	2 / 0.3	None	ADA (13.7)	No / 18.8

Abbreviations: ADA, adalimumab; AZA, azathioprine; CS, corticosteroids; CsA, cyclosporine A; ETN, etanercept; F, female; IFX, infliximab; M, male; MTX, methrotexate; NA, not available.

***Appendix 1.* Complete list of investigators.**

Inés Rita GALÉ-ANSO (Hospital Universitario Miguel Servet, Zaragoza, Spain); Pilar BERNABÉU, Mercedes JUSTE (Hospital Universitario San Juan de Alicante, Alicante, Spain); Elena COLINO-GIL (Mother and Child Insular University Hospital, Las Palmas, Spain); Federico MARTINÓN-TORRES, Antonio JUSTICIA (University Clinical Hospital of Santiago, Santiago de Compostela, Spain); Juan Carlos LÓPEZ-ROBLEDILLO, Daniel CLEMENTE (Hospital Infantil Universitario Niño Jesús, Madrid, Spain); Sara MURÍAS, Rosa ALCOBENDAS, Ana MÉNDEZ-ECHEVARRÍA, Walter Alfredo GOYCOECHEA-VALDIVIA, Gerardo PRIETO (Hospital La Paz, Madrid, Spain); Laura FERNÁNDEZ-SILVEIRA, María Isabel GONZÁLEZ-FERNÁNDEZ, Anabel PIQUERAS (University Hospital La Fe, Valencia, Spain); Olaf NETH, Alejandro RODRÍGUEZ-MARTÍNEZ, Marta MELÓN, Lola FALCÓN (Hospital Universitario Virgen del Rocío, Sevilla, Spain); Laura CRESPO-VALDERRÁBANO (Hospital Universitario Río Hortega de Valladolid, Valladolid, Spain); María Jesús RÚA (Hospital de Cruces, Barakaldo, Spain).
